# Dual role of *ramR* mutation in enhancing immune activation and elevating eravacycline resistance in *Klebsiella pneumoniae*

**DOI:** 10.1002/imo2.39

**Published:** 2024-11-09

**Authors:** Wei Yu, Peiyao Jia, Xiaobing Chu, Shengjie Li, Xinmiao Jia, Ying Zhu, Xiaoyu Liu, Yingchun Xu, Qiwen Yang

**Affiliations:** ^1^ Department of Clinical Laboratory State Key Laboratory of Complex Severe and Rare Diseases, Peking Union Medical College Hospital, Chinese Academy of Medical Sciences and Peking Union Medical College Beijing China; ^2^ Graduate School, Peking Union Medical College, Chinese Academy of Medical Sciences Beijing China; ^3^ Biomedical Engineering Facility, National Infrastructures for Translational Medicine, Institute of Clinical Medicine & Peking Union Medical College Hospital, Chinese Academy of Medical Sciences and Peking Union Medical College Beijing China; ^4^ Center for Bioinformatics National Infrastructures for Translational Medicine, Institute of Clinical Medicine & Peking Union Medical College Hospital, Chinese Academy of Medical Sciences and Peking Union Medical College Beijing China; ^5^ Key Laboratory of Pathogen Infection Prevention and Control (Peking Union Medical College), Ministry of Education Beijing China

**Keywords:** AcrAB‐TolC efflux pump, eravacycline resistance, immune activation, *Klebsiella pneumoniae*, lipid A modification, ramR

## Abstract

The rise of *Klebsiella pneumoniae* resistant to last‐resort antimicrobials poses an urgent threat to global health. The *ramR*‐*ramA* regulatory system critically influences drug resistance by regulating the AcrAB‐TolC efflux pump, which also plays a crucial role in the pathogenicity of *K. pneumoniae*. However, the mechanism of the *ramR*‐*ramA* system on bacteria‐host interaction remains unclear. To determine how specific mutations in *ramR* influence eravacycline (ERV) resistance and their impact on the immune activation capabilities of *K. pneumoniae*, thereby highlighting potential targets for therapeutic intervention, we performed genetic sequencing to identify mutations in *ramR*. Then, the CRISPR‐Cas9 technology was employed to construct specific *ramR* mutations into *K. pneumoniae*, which were then subjected to phenotypic and functional assays in both in vitro and in vivo (mouse models, macrophage, and blood‐killing experiment) settings. *ramR* L58P and F165L genetic alterations disrupt the binding affinity of RamR to the *ramA* promoter, thereby upregulating the efflux pump expression and increasing ERV minimum inhibitory concentration values up to 64‐fold compared to the wild‐type. Concurrently, these mutations modulate lipid A structure by increasing 2‐hydroxy fatty acid chain abundance. In mouse models, *ramR* L58P and F165L mutants showed lower bacterial burden in organs (spleen, lung, and kidney) 6 h post‐infection, and are fast cleared in 48 h. Furthermore, despite lower intracellular bacterial loads, *ramR* L58P and F165L mutants induce heightened pro‐inflammatory cytokine responses in macrophages and elevate systemic cytokine levels (interleukin [IL]2, IL4, IL6, IL12, interferon‐α, and interferon‐γ) in human blood co‐culture experiments. This study illuminates the critical role of *ramR* mutations in conferring ERV resistance and enhancing immune responses in *K. pneumoniae*. The dual impact of these mutations on both antimicrobial resistance and immune activation not only underscores the challenges in treating infections but also advocates for heightened surveillance and innovative strategies to counteract the emerging threat of antimicrobial‐resistant *K. pneumoniae*.

## Highlights

- *ramR* mutation L58P and F165L reduce the binding affinity of RamR with the *ramA* promoter region, thus dissolving the inhibitory effect of *ramR* on *ramA* expression and leading to AcrAB‐TolC/OqxAB efflux pump's high expression.
- *ramR* mutations L58P and F165L increase *K. pneumoniae* eravacycline resistance up to 64‐fold.
- These two mutations increase the 2‐hydroxy fatty acid chain proportion of lipid A in *K. pneumoniae* and reduce the growth rates of mutated strains.
- When infected with *ramR* L58P and F165L mutants, a faster organ/blood clearance rate and lower mortality rate are found in mouse models.
- Elevated pro‐inflammatory cytokine responses induced by *ramR* L58P and F165L mutants in macrophages and human blood.
- A dual function of *ramR* L58P and F165L in elevating eravacycline resistance and host immune response is currently studied.

## Graphical abstract

The *ramR* mutations L58P and F165L weaken the binding between RamR and the *ramA* promoter, lifting RamR's suppression of *ramA* expression and resulting in the overexpression of the AcrAB‐TolC and OqxAB efflux pumps. Thus, these mutations enhance *Klebsiella pneumoniae*'s resistance to eravacycline, up to 64‐fold. Additionally, they alter the lipid A composition by raising the proportion of 2‐hydroxy fatty acids. In mouse infection models, strains with the *ramR* L58P and F165L mutations exhibit faster clearance from organs and blood and are associated with lower mortality rates. Furthermore, these mutations trigger stronger pro‐inflammatory cytokine responses in macrophages and human blood. The dual role of *ramR* L58P and F165L mutations in boosting both eravacycline resistance and host immune response is currently being investigated.

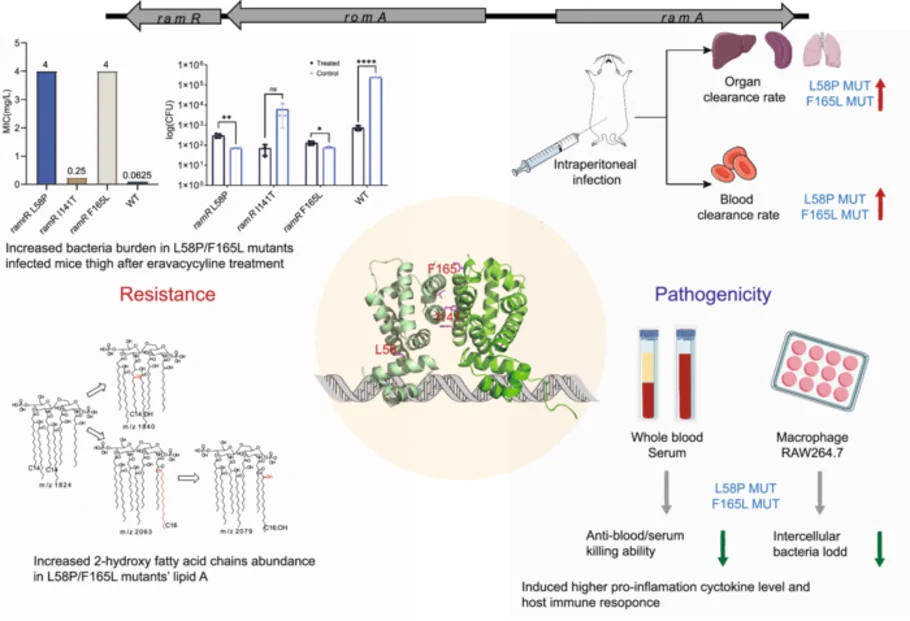

## 1 INTRODUCTION

Multidrug resistance (MDR) in gram‐negative bacteria, particularly in *Klebsiella pneumoniae*, poses a significant threat to public health, and the use of antimicrobials in agriculture, especially in livestock and poultry, has accelerated the emergence of antimicrobial‐resistant bacteria [1, 2]. Of particular concern is the rapid and extensive development of resistance to commonly used clinical antimicrobials in *K. pneumoniae*. Furthermore, the emergence of carbapenemase‐resistant hypervirulence *K. pneumoniae* (CR‐hvKP) exacerbates the severity of this issue [3, 4]. The emergence and spread of antimicrobial resistance genes in animal‐source bacteria, which may accumulate through foodborne transmission, also arouse concern [5]. The management of infections caused by the CR‐hvKP often necessitates the use of last‐resort antimicrobials, including colistin and tigecycline [6]. Nevertheless, the rapid emergence of tigecycline‐resistant strains of *K. pneumoniae* on a global scale following its introduction into clinical practice [7, 8, 9], coupled with the potential for healthcare‐associated misuse of antimicrobials, poses a significant challenge to effective treatment strategies [10].

Eravacycline (ERV), a novel antimicrobial, is a fully synthetic fluorocycline of the tetracycline class developed for the medication for serious infections. *In vitro* studies showed that ERV had a potential activity against a wide range of clinically important gram‐negative bacteria, including ESBL‐producing and carbapenem‐resistant *Enterobacteriaceae* [11]. Notably, eravacyline has activity in tetracycline‐resistant bacteria since it can resist the major tetracycline‐specific resistance mechanisms, such as ribosomal protection protein (TetM) and efflux pump gene *adeB* variation in *Acinetobacter baumannii* [12, 13]. However, ERV‐nonsusceptible strains were detected even though the ERV has not been used clinically [14].

Up to the present, the main tetracycline‐resistant mechanism is believed to be related to RND‐efflux pumps including AcrAB‐TolC/OqxAB pumps. The expression level of these pumps can be regulated by RamA, RamR, OqxR, and AcrR [15, 16]. RamA encoded by *ramA* acts as a global transcriptional activator for AcrAB‐TolC, while *ramR* encodes the local repressor RamR for *ramA* [17, 18]. Additionally, the transcription level of AcrAB/OqxAB is also regulated by its local repressor AcrR and OqxR, which are encoded by *acrR* and *oqxR*, respectively. Previous studies have reported that mutations in these regulation genes can lead to the upregulation of RND‐efflux pumps, thus contributing to tigecycline resistance [14, 19, 20]. However, it is still unknown which regulator gene mutations directly promote ERV resistance in *K. pneumoniae*.

Except for antimicrobial resistance, AcrAB‐TolC efflux pumps have also been reported relating to bacterial pathogenicities [21]. In particular, in *Shigella flexneri* both *acrA* and *acrB* genes contribute to the invasion of epithelial cells [22]. In the infectious process of the adherent‐invasive *Escherichia coli*, the AcrAB‐TolC is crucial for intramacrophage survival [23, 24]. In a mouse model, both *acrA*‐knockout and *tolC*‐knockout strains presented significantly lower bacterial loads in organs than *Enterobacter cloacae* wild‐type (WT) [25]. The virulence of the AcrB D408A mutant was attenuated in vivo in mouse and *Galleria mellonella* models, and this mutant showed significantly reduced invasion into intestinal epithelial cells and macrophages in vitro in *Salmonella enterica Serovar* Typhimurium [26]. Moreover, the AcrAB efflux pump helps *K. pneumoniae* to resist innate immune defense, and the AcrB knockout exhibited a reduced capacity to cause pneumonia in mice, compared to the WT [27]. One study reported that RamA‐mediated lipid A structural modification contributes to increased survival under host immune pressure [28]. Though *ramA* is reported closely related to lipid A biosynthesis, and various mutations have been implicated in tigecycline/ERV resistance [20, 29], few have been functionally validated.

In this study, 500 clinical strains that had not been exposed to ERV before were collected to evaluate their ERV susceptibility profiles from 5 hospitals in China. Several important mutations in *ramR* were detected in the present study. Then, a CRISPR‐Cas system was induced in a CR‐hvKP to define the effects of these mutations on ERV resistance, lipid A biosynthesis and structural modification, bacterial virulence, and host‐microbiome immune reaction in CR‐hvKP. The main purpose of our study was to determine the specific point mutation that influences the ERV‐resistance and pathogenicity in *K. pneumoniae*. By pinpointing specific genetic mutations associated with ERV resistance and pathogenic behavior in *K. pneumoniae*, this research may pave the way for the development of targeted diagnostic tests and therapeutic strategies.

## 2 RESULTS

### 2.1 Efflux pump overexpression contributes to ERV resistance in *K. pneumoniae*

**Figure 1:**
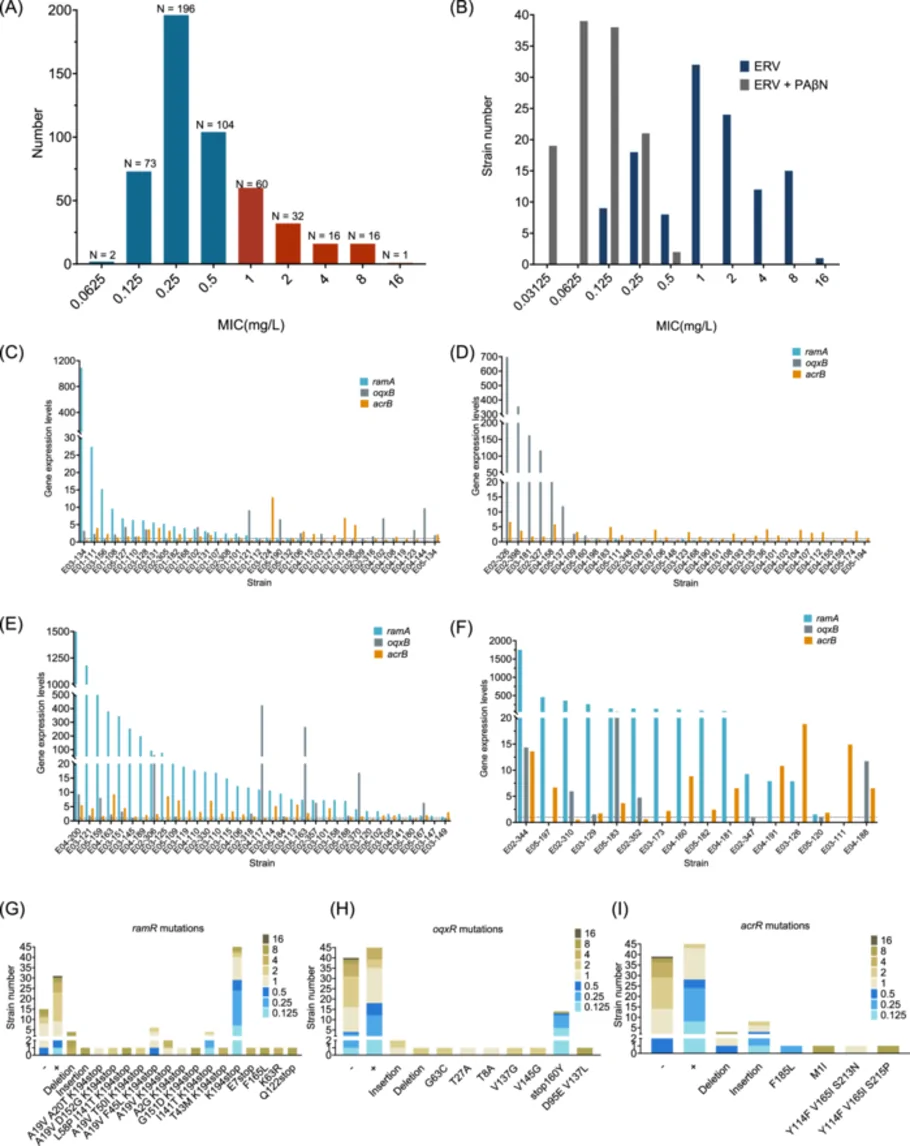
Distribution of eravacycline minimum inhibitory concentrations (MICs) and expression levels of efflux pump‐related genes in *Klebsiella pneumoniae*. (A) MIC distribution of eravacycline in 500 clinical *K. pneumoniae* isolates, showing variability in resistance levels. (B) Shift in MIC distribution upon the addition of the efflux pump inhibitor PAβN, indicating a reduction in eravacycline resistance. (C–F) Expression levels of efflux pump genes *ramA*, *acrB*, and *oqxB*, across different MIC categories; isolates with MIC ≤ 1 μg/mL (C), isolates with MICs of 2–4 μg/mL (D, E), and isolates with MIC ≥ 8 μg/mL (F). (G–I) The MIC distributions and counts of isolates with mutations in regulatory genes of efflux pumps, and different colors indicate different MIC values; *ramR* mutations (G), *oqxR* mutations (H), and *acrR* mutations (I), illustrating the correlation between specific genetic alterations and eravacycline resistance profiles. ERV, eravacycline.

Of all the 500 clinical *K. pneumoniae* isolates, ERV minimal inhibitory concentration (MIC) values ranged from 0.0625 to 16 mg/L, and the susceptible rate was 75.0% according to the Food and Drug Administration (FDA) criteria (≤0.5 mg/L) (Figure 1A). To investigate the ERV resistance mechanism in *K. pneumoniae*, we randomly selected 119 strains with MIC ranging from 0.125 to 16 mg/L (Table S1) to conduct the efflux pump inhibitors (EPIs) experiment. After being exposed to PAβN, 97.6% of strains restored susceptibilities (Figure 1B) (all strains could grow when only 50 μM PAβN was added, see Figure S1).

We then tested efflux pump genes *acrB*, *oqxB*, and the regulator gene *ramA* expression levels, and target genes’ expression levels were compared within 3 groups divided according to the ERV breakpoint and MIC values (≤0.5 mg/L, 1–4 mg/L, ≥8 mg/L). Expression levels of *ramA*, *oqxB*, and *acrB* exhibited significant differences among the 3 groups (*p* < 0.05) (Table 1). Gene expression levels of different MIC groups were illustrated in Figure 1C–F. In the ERV‐nonsusceptible group, overexpression of *acrB* and/or *oqxB* was constantly observed with high‐level *ramA* expressions (Figure 1D–F). All strains whose ERV MIC ≥ 8 mg/L exhibited highly expressed either *acrB* or *oqxB*.

**Table 1:** Relations between gene expression level and eravacycline MIC of 119 *Klebsiella pneumoniae* clinical isolates.

	Gene expression level (medium [IQR])					
Gene	MIC ≤ 0.5 mg/L	1 mg/L ≤ MIC ≤ 4 mg/L	MIC ≥ 8 mg/L	*H*	*ν*	*p* value
*ramA*	1.23 [0.00, 5.25]	1.77 [0.00, 11.70]	101.60 [7.87, 234.65]	9.711	2	0.0008
*oqxB*	1.37 [0.70, 3.23]	0.40 [0.00, 6.79]	0.46 [0.00, 5.64]	6.896	2	0.032
*acrB*	1.44 [0.74, 2.36]	2.04 [1.13, 3.66]	5.10 [1.75, 10.31]	12.857	2	0.002

*Note*: Continuous variables were assessed for normality using the Shapiro–Wilk test. Variables that followed a normal distribution were presented as mean ± standard deviation, while those that did not follow a normal distribution were presented as median and interquartile range (IQR). Variances were not equal; thus, the non‐parametric Kruskal–Wallis test was used.

Abbreviations: H, Kruskal–Wallis *H*‐test value; MIC, minimum inhibitory concentration; ν, degree of freedom.

### 2.2 Mutations in efflux pump regulator genes (*ramR*, *oqxR*, and *acrR*) lead to efflux pump overexpression and ERV resistance

To explain the reason for efflux pump overexpression, we sequenced several main regulator genes of efflux pumps. The frequency of point mutations in *ramR* was much higher than in *oqxR* and *acrR* (Table S2), and mutations leading to amino acid alterations in RamR may cause ERV resistance more probably (Figure 1G–I). Except for deletions, insertions, and premature termination in *ramR*, point mutations in *ramR* could dramatically promote the expression level of *ramA* with the highest up to 1752.26‐fold. A statistical difference in ERV MIC values was acquired between the WT and *ramR*/*oqxR* mutants (*p* < 0.05). Besides, we tested the tigecycline resistance‐associated genes, and among 119 *K. pneumoniae* isolates, no *tet(M), tetL, or tetX* genes were found. The *tet(A)* gene was detected in 71 isolates, all of which were WT. And the *rpsJ* WT was detected in 114 isolates.

**Figure 2:**
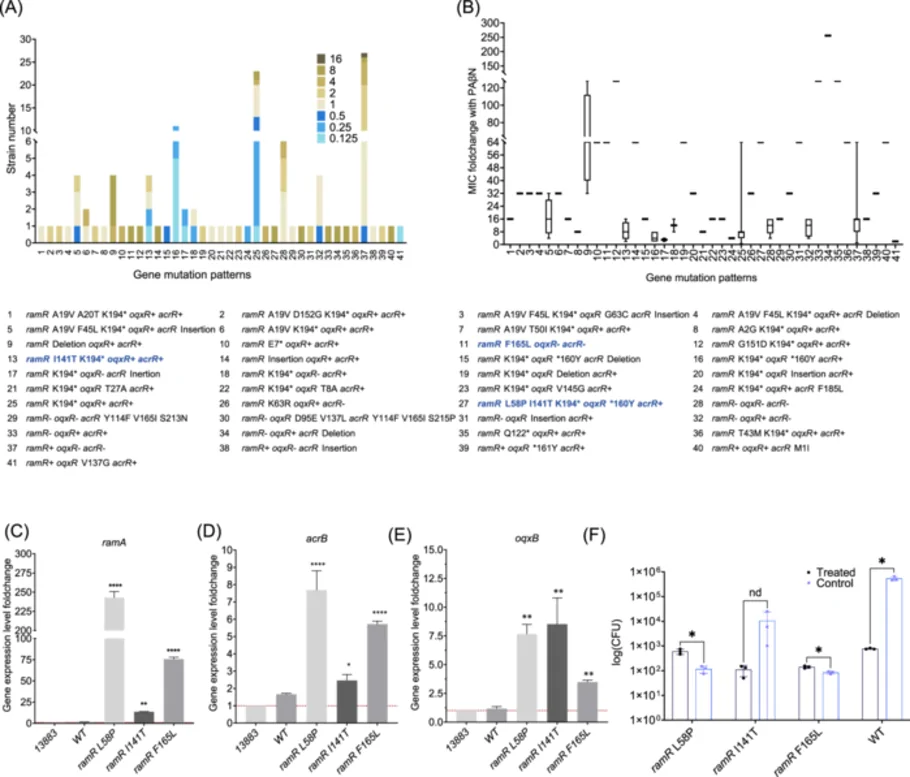
Analysis of gene mutations associated with eravacycline resistance in *K. pneumoniae*. (A) The distribution of 41 detected gene mutation patterns in relation to the strain's MIC and corresponding counts. The absence of regulatory genes *ramR*, *oqxR*, and *acrR* is linked to increased resistance to eravacycline. (B) Fold reduction in MIC after the addition of PAβN. Strains with higher resistance levels are more significantly affected by efflux pump inhibition. Blue‐marked gene mutation combinations represent mutations highly correlated with resistance based on statistical analysis. (C–E) Gene expression levels of *ramA*, *acrB*, and *oqxB* in genetically modified strains constructed by CRISPR‐Cas9. (F) Variation in bacterial load within mouse muscle tissue post‐injection with *K. pneumoniae*, comparing the effects of eravacycline treatment. Strains harboring resistance mutations show an increasing trend in bacterial load after antimicrobial treatment, suggesting an adverse effect on the efficacy of eravacycline in controlling infections with resistant strains. *p < 0.05, **p < 0.01, ***p < 0.001, ****p < 0.0001, MIC, minimum inhibitory concentration; nd, no difference.

We defined 41 mutation patterns (the combination of *ramR, acrR*, and *oqxR* gene mutations) in 119 *K. pneumoniae* isolates in the present study. When *K. pneumoniae* isolates did not harbor repressor genes or carried premature repressor proteins due to nonsense mutations in their coding genes, the expression level of efflux pumps would be upregulated, and MIC values of ERV were more likely high. Isolates harboring specific mutation patterns (*ramR* F165L *oqxR*‐ *acrR*‐, *ramR* G151D K194stop *oqxR* WT *acrR* WT, *ramR* K63R *oqxR* WT *acrR*‐, *ramR* L58P I141T K194stop *oqxR* stop160Y *acrR* WT, *ramR*‐ *oqxR* D95E V137L *acrR* Y114F V165I S215P, *ramR* T43M K194stop *oqxR* WT *acrR* WT, and *ramR* WT *oqxR* WT *acrR* M1I) obtained higher MIC values (4–8 mg/L) than those who did not (Figure 2A). Additionally, all the resistance caused by these mutation patterns could be reversed by PAβN, making MICs drop by 32–256‐fold (Figure 2B).

To determine which mutations can cause ERV resistance, linear regression analysis was performed in our study to evaluate the portion of each mutation type taking in the elevation of ERV MIC value. Eight mutation types were included in the model, and 6 of them showed statistical significance (Table S3). Importantly, point mutations *ramR* L58P, I141T, and F165L could considerably increase ERV MIC values (mutation pattern 27, 13, 11 in Figure 2).

### 2.3 *ramR* mutations increase antimicrobial resistance in vitro and in vivo

To investigate the impact of point mutations (*ramR* L58P, I141T, and F165L) on *K. pneumoniae* resistance, especially against ERV, we generated three mutants based on AZJ065. RT‐qPCR showed that the expression level of *ramA* increased more drastically in *ramR* L58P (242.8‐fold) and *ramR* F165L (75.6‐fold) mutants than in *ramR* I141T (13.6‐fold). This overexpression led to highly expressed *acrB* and *oqxB* (Figure 2C–E). The ERV MIC for *ramR* L58P and *ramR* F165L mutants increased 64‐fold to 4 mg/L, and this effect was reversible when using PAβN (50 µM) (Table 2). Conversely, *ramR* I141T mutants exhibited only marginal changes in their MICs, reaching 0.25 mg/L. The MICs of tetracycline, tigecycline, minocycline, and levofloxacin also increased significantly in *ramR* L58P and F165L mutants (tetracycline: from 1 to 16 mg/L, tigecycline: from 0.125 to 4 mg/L, minocycline: from 2 to 16 mg/L, and levofloxacin: from 1 to >4 mg/L and 4 mg/L, respectively). Moreover, *ramR* L58P and *ramR* F165L mutants displayed elevated MIC values for several clinically commonly used antimicrobials (Ceftazidime, Levofloxacin, and Cefoxitin).

**Table 2:** In vitro susceptibilities of the WT and *ramR* mutants to eravacycline and other antimicrobials.

Antibiotic agents	MIC (mg/L)			
	WT	WT‐*ramR* L58P	WT‐*ramR* I141T	WT‐*ramR* F165L
ERV	0.0625	4	0.25	4
ERV + PAβN	<0.0625	0.5	0.5	0.5
TET	1	16	1	16
TGC	0.125	4	0.5	4
MH	2	16	2	16
LEV	1	>4	1	4
COL	≤1	≤1	≤1	≤1
ETP	>4	>4	>4	>4
FOX	16	>16	16	>16
IMP	16	>16	16	16
AXO	>8	>8	>8	>8
P/T	>64/4	>64/4	>64/4	>64/4
TAZ	16	>16	16	>16
CZA	0.5/4	0.5/4	0.25/4	1/4
MEM	16	>16	>16	>16
C/T	>16/4	>16/4	>16/4	>16/4
FEP	>16	>16	>16	>16
AMI	≤8	≤8	≤8	≤8
AZT	>8	>8	>8	>8
IMK	0.25/4	≤0.125/4	0.25/4	≤0.125/4

Abbreviations: AMI, Amikacin; AXO, Ceftriaxone; AZT, Aztreonam; COL, Colistin; C/T, Ceftolozone‐tazobactam; CZA, Ceftazidime/avibactam; ERV, Eravacycline; ETP, Ertapenem; FEP, Cefepime; FOX, Cefoxitin; IMK, Imipenem/Relebactam; IMP, Imipenem; LEV, Levofloxacin; MEM, Meropenem; MH, Minocycline; PAβN, Phenylalanine‐arginine β‐naphthylamide; P/T, Piperacillin/Tazobactam; TET, Tetracycline; TGC, Tigecycline; TAZ, Ceftazidime.

Furthermore, a thigh infection mouse model was utilized to evaluate the resistance level of strains in vivo. Compared to the control group, significantly lower bacterial loads were observed in mice infected with the susceptible strains (WT and *ramR* I141T mutant) after ERV treatment (Figure 2F). However, in ERV‐resistant mutants (*ramR* L58P and *ramR* F165L), bacterial burdens increased following ERV treatment.

### 2.4 *ramR* mutations increase gene expression levels involved in lipid A synthesis and structural modification

**Figure 3:**
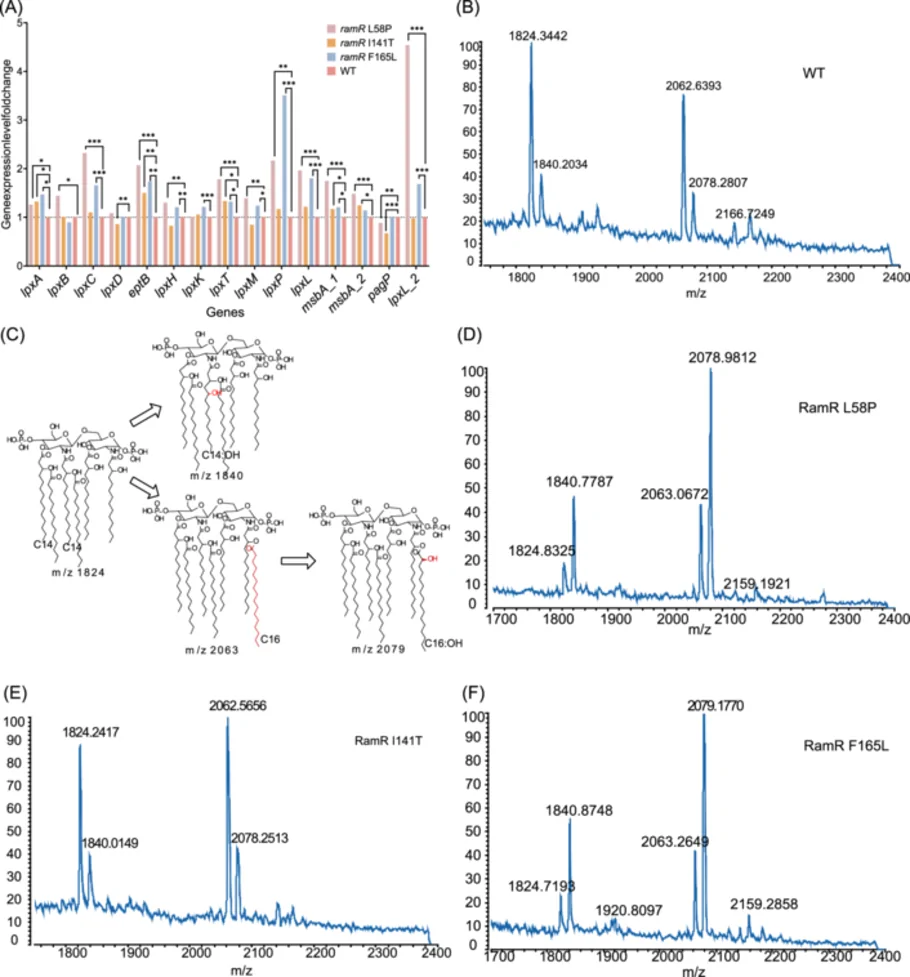
Expression and mass spectrometry analysis of lipid A in *K. pneumoniae*. (A) Expression levels of genes involved in the lipid A biosynthetic pathway in mutant strains. (B) Mass spectrum of wild‐type *K. pneumoniae* lipid A showing prominent molecular ions indicative of a predominance of hexa‐acylated species with a mass‐to‐charge ratio (m/z) of 1824, corresponding to a structure of two glucosamines, two phosphates, four 3‐OH‐C14 groups, and two C14 groups. A second hexa‐acylated species with an m/z of 1840 was observed, consisting of one C14: OH group, one C14 group, four 3‐OH‐C14 groups, two glucosamines, and two phosphates. (C) Chemical structure of lipid A and its modified forms, with corresponding m/z ratios indicating the presence of hexa‐acylated species and their modifications by the addition of a palmitate or ‐OH group. (D–F) Mass spectra for *ramR* L58P, *ramR* F165L, and *ramR* I141T mutants depicting variations in lipid A profiles. The mutants *ramR* I141T displayed mass spectrometric graphs similar to the wild type, primarily featuring the m/z 1824 hexaacylated lipid A and the m/z 2063 species (hexaacylated lipid A with additional palmitate). Lipid A from *ramR* L58P and *ramR* F165L mutants exhibited significant peaks at m/z 1840 and m/z 2078, suggesting a higher proportion of lipid A modified with a 2‐hydroxy fatty acid chain, potentially affecting host‐microbe immune interactions. Simplified, the wild‐type lipid A predominantly consists of hexa‐acylated species. Differences observed in mutants may impact the abundance of various lipid A species and their immune recognition. *p < 0.05, **p < 0.01, ***p < 0.001, ****p < 0.0001.

Previous work in *K. pneumoniae* showed that *ramA* directly binds and activates the expression of *lpxC*, *lpxL‐2*, and *lpxO* gene promoters [28]. To further elucidate the impact of *ramR* mutant strains on virulence, we investigated whether *ramR* point mutations could affect genes involved in the lipid A biosynthesis pathway (*lpxA‐D, lpxH, lpxK, lpxL, plxL‐2, lpxM, lpxP, lpxT, msbA‐1, msbA‐2, pagP*, and *etpB*) [30, 31]. Except for *lpxC* and *lpxL‐2*, the expression levels of *lpxH, lpxL, lpxM, lpxT, msbA‐1, etpB* were also significantly elevated in both *ramR* L58P and F165L mutants (Figure 3A). Specifically, the expression level of *lpxL‐2* was remarkably increased by 4.54‐fold in the *ramR* L58P mutant. Additionally, *ramR* L58P and *ramR* F165L mutants showed elevated expression of *lpxB* (1.44‐fold) and *lpxK* (1.24‐fold), respectively.

To further investigate the lipid A structural modification, the mass spectrum was conducted. The lipid A moiety alteration was confirmed in *ramR* L58P and *ramR* F165L mutants, where peaks at *m/z* 1840 and *m/z* 2078 were found elevated compared to the WT (Figure 3B–F). The *ramR* I141T mutant showed a similar mass spectrometry graph to the WT. Previous work in *K. pneumoniae* showed that these peaks correspond to hydroxy hexa‐acylated or palmitate lipid A species [32].

### 2.5 *ramR* mutation reduces binding affinity with the *ramA* promoter region

**Figure 4:**
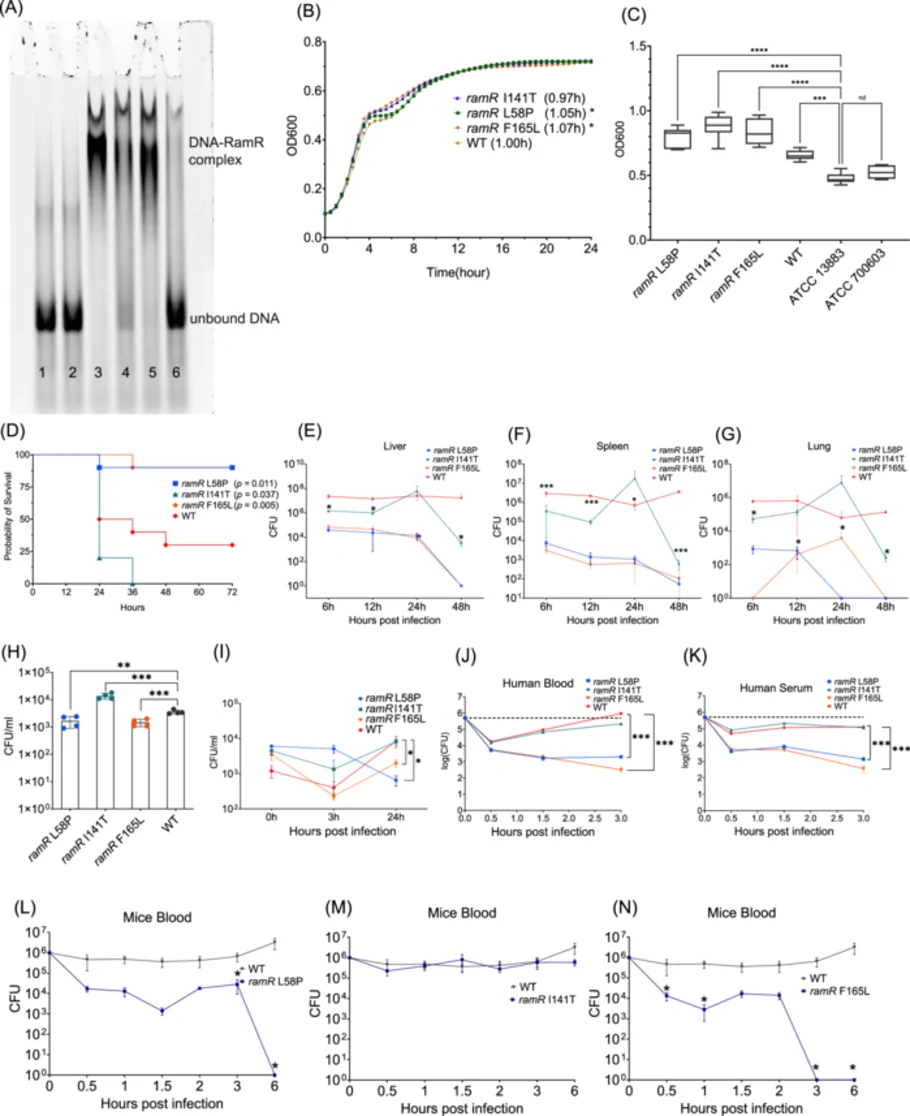
Assessment of fitness cost, biofilm formation, and in vivo virulence of wild‐type (WT) and mutant strains of *K. pneumoniae*. (A) Electrophoretic mobility shift assay using purified RamR protein. Lane 1. null, Lane 2. BSA, Lane 3. WT, Lane 4. RamR F165L, Lane 5. RamR I141T, Lane 6. RamR L58P. WT and RamR I141T decreased the migration of promotor DNA of *ramA*. RamR L58P showed no binding ability to promote the DNA of *ramA*. (B) Comparison of growth rate (μ) between mutant strains and WT, with *ramR* L58P and *ramR* F165L exhibiting significantly higher growth rates, indicating a fitness cost due to the mutations. (C) Biofilm formation capability of mutant strains compared to WT, demonstrating significantly higher biofilm production in mutants. ATCC 13883 and ATCC 700603 serve as quality control strains. (D) Kaplan–Meier survival curves of mice infected with WT or its *ramR* mutants for 72 h. *p* values were determined by the log rank (Mantel‐Cox) test. (E–G) Bacterial load trends in liver, spleen, and lung at different time points post‐intraperitoneal infection in mice, with data from three mice per time point. (H) Intracellular bacterial load in RAW264.7 cells after 2 h of co‐incubation, each point representing a biological replicate. (I) Bacterial survival within RAW264.7 cells over time, starting from 2 h post co‐incubation (0 h). No significant differences in initial intracellular bacterial load, but after 24 h, *ramR* L58P and *ramR* F165L numbers were significantly lower than WT, while *ramR* I141T showed no difference. (J, K) Bacterial load changes over time when strains are co‐incubated with healthy human whole blood and serum. *ramR* I141T showed consistent resistance to blood/serum bactericidal activity compared to WT, whereas *ramR* L58P and *ramR* F165L showed a significant decrease in bacterial count after 3 h. (L–N) Bacterial load variations in the bloodstream of mice post septicemia by *ramR* L58P, *ramR* I141T, and *ramR* F165L mutants. Consistent with in vitro findings, *ramR* L58P and *ramR* F165L showed a significant reduction in blood bacterial load after 1 h, while *ramR* I141T resisted blood bactericidal activity, persisting at the initial inoculum level over time akin to wild type. *p < 0.05, **p < 0.01, ***p < 0.001, ****p < 0.0001.

To study the interaction of RamR with the *ramA* promoter, we produced RamR protein as a recombinant His6‐RamR in *E. coli* (BL21‐DE3) with a theoretical molecular mass of 23,320 Da. The protein has been subjected to a series of purification processes for homogeneity, including affinity chromatography, ion exchange chromatography, and size exclusion chromatography. We conducted an electrophoretic mobility shift assay (EMSA) with a 95 bp DNA fragment containing the putative RamR binding site of the *ramA* promoter. As shown in Figure 4A, a shifted band, indicative of DNA fragment binding, is anticipated for both the WT RamR and the *ramR* I141T mutant variant. However, the *ramR* L58P mutation appears to result in a complete loss of DNA binding capability. Additionally, the *ramR* F165L mutation in the RamR protein is observed to attenuate its DNA binding affinity.

### 2.6 *ramR* mutants affect growth rate in vitro and virulence in vivo

Lipid A, an integral component of bacterial structure, plays a crucial role in bacterial growth rate regulation. As expected, *ramR* L58P and *ramR* F165L showed lower growth rates (1.05 and 1.07 h, respectively), with their growth curves shifted to the right (Figure 4B). While the biofilm formation ability was enhanced in *ramR* mutants (Figure 4C).

To assess the virulence of *ramR* mutants, mice were intraperitoneally injected and monitored for 72 h (Figure 4D). The lethal capabilities of *ramR* L58P and F165L mutants were significantly lower than those of the WT strain (*p* = 0.011, *p* = 0.005, respectively), while *ramR* I141T mutant showed a higher mortality rate (*p* = 0.037).

### 2.7 Mutations in *ramR* impeded bacteria accumulation in mice organs

Subsequently, we compared the ability of *ramR* mutants and the WT to cause systemic dissemination and organ failure by performing a time‐course organ bacterial clearance experiment. Consistent with the observed mortality rates of each strain, the *ramR* L58P and F165L mutants, which showed decreased mortality rates, exhibited significantly lower initial bacterial loads that were gradually eliminated from all organs, except in the lung, where the bacterial load of *ramR* F165L increased from 6 to 24 h post‐infection followed by a drastic decrease from 24 to 48 h (Figure 4E–G). In contrast, the WT strain showed higher bacterial loads in all organs and was more difficult to eliminate compared to the mutants. The bacterial levels in each organ for all mutants decreased 24 h after infection. These results showed that the WT (a CR‐hvKP) exhibited bacterial persistence in vivo, and the lipid A modification in *ramR* mutants impeded the accumulation and survival of *K pneumoniae* in the liver, spleen, and lungs of mice.

### 2.8 *ramR* mutations affected macrophage activation and phagocytosis

To further verify that *ramR*‐mediated lipid A modification could influence macrophage phagocytosis, we co‐cultured mutants and macrophages RAW264.7. We found that macrophages infected with *ramR* L58P and F165L mutants exhibited significantly reduced intercellular bacterial counts compared to the WT (Figure 4H). Conversely, *ramR* I141T mutant displayed enhanced phagocytosis, with increased intercellular bacterial counts.

Subsequently, to assess the intracellular survival capability of bacteria post‐phagocytosis, we removed the bacterial‐containing media and extended the incubation time. No significant differences in intracellular bacterial counts were observed after phagocytosis among the mutant strains. After 24 h of intracellular incubation, both *ramR* L58P and *ramR* F165L mutants exhibited substantially lower bacterial loads compared to the WT, while *ramR* I141T mutants showed similar bacterial burden to the WT (Figure 4I).

### 2.9 *ramR* L58P and F165L weaken *K. pneumoniae* anti‐blood/serum killing in vitro and in vivo

In organs and macrophages, lipid A modification reduces the strain's ability to resist host immune clearance. A similar phenomenon exists in peripheral blood, as the *ramR* L58P and *ramR* F165L mutants showed a continuous decline in bacterial counts and exhibited significantly lower survived numbers than the WT (*p* < 0.001) when incubated with whole blood (Figure 4J). In serum, all strains experienced reduced counts after incubation. The *ramR* F165L and L58P mutants showed significantly lower survival compared to the WT, while the *ramR* I141T mutant did not significantly differ (Figure 4K).

Similarly, a consistent trend was observed in a mouse bloodstream infection model in vivo. Following bloodstream infections, *ramR* L58P and F165L mutants exhibited a sharp decrease in blood counts at 0.5 h (Figure 4L–N). Complete clearance was observed at 3 and 6 h post‐infection for *ramR* F165L and *ramR* L58P mutants, respectively. Conversely, the blood bacterial load of *ramR* I141T remained comparable to that of the WT, demonstrating strong resistance to blood‐killing actions.

### 2.10 *ramR* L58P and F165L enhanced pro‐inflammation cytokine response

**Figure 5:**
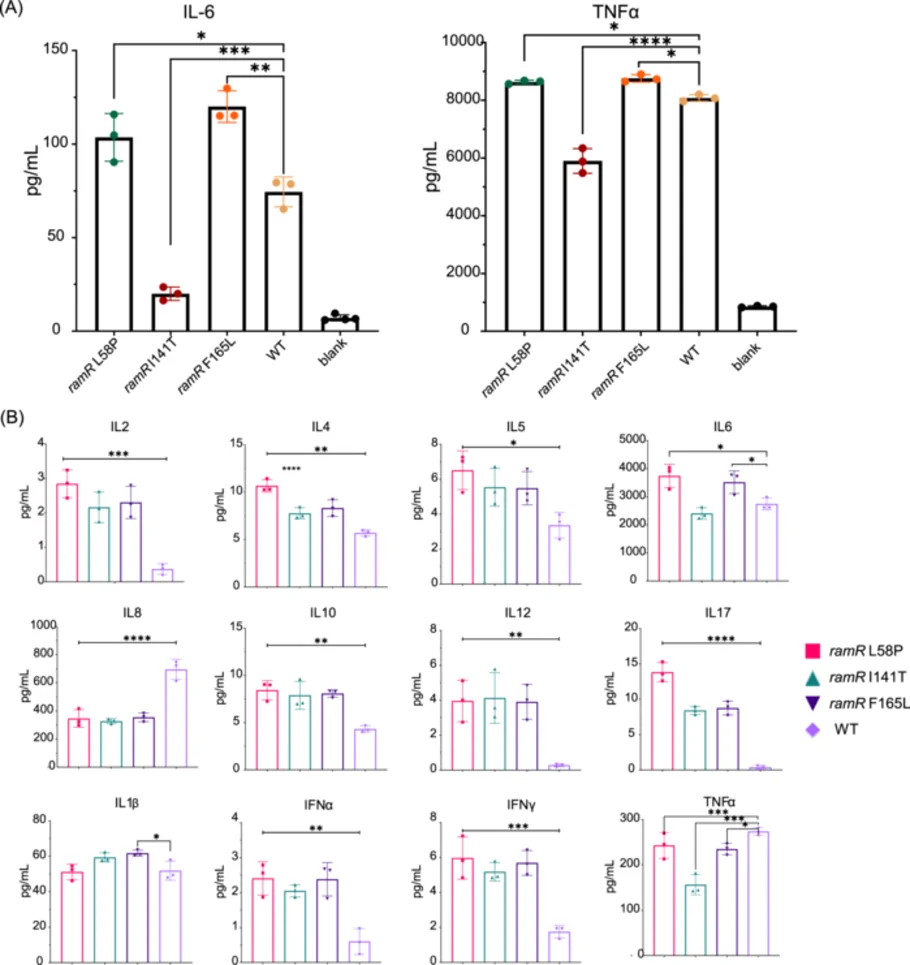
Cytokine levels after co‐incubated with *K. pneumoniae*. (A) IL‐6 and TNFα levels released into the medium by RAW264.7 cells after 2 h of co‐incubation with the strains. (B) Pro‐inflammatory cytokine levels (pg/mL) in the blood after 3 mutants co‐incubated with blood in vitro. *p < 0.05, **p < 0.01, ***p < 0.001, ****p < 0.0001. IL, interleukin; TNF, Tumor necrosis factor.

To define the activation of macrophages, we collected supernatants from co‐cultures of bacteria and macrophages to assess the levels of the MyD88‐dependent cytokines tumor necrosis factor alpha (TNFα) and interleukin‐6 (IL6). Macrophages infected with *ramR* L58P and F165L mutants exhibited higher levels of the TNFα and IL6 release compared to the WT (Figure 5A,B), whereas *ramR* I141T mutants displayed lower cytokine levels than the WT. To further investigate the weakened blood/serum bactericidal ability of *ramR* L58P and *ramR* F165L mutants, we assessed the cytokine levels in whole blood following bacterial infection. As expected, co‐culturing with *ramR* L58P and *ramR* F165L mutants resulted in a significant elevation of pro‐inflammatory cytokine levels in the blood (IL2, IL4, IL6, IL12, interferon [IFN]‐α, and IFN‐γ) (Figure 5).

## 3 DISCUSSION

ERV, an alteration for tigecycline, was approved for complicated intra‐abdominal infections (cIAI) treatment and showed good in vivo activity against *Enterobacteriaceae*, and *Acinetobacter baumannii* (including MDR). Our study broadens the multicentral reports of ERV susceptibility in *K. pneumoniae* from China mainland. Compared with surveillance in Taiwan from 2017 to 2020, the susceptible rate of ERV against *K. pneumoniae* was quite lower in our study (84% in Taiwan) [33]. In another study in South China, 83% carbapenem‐resistant *K. pneumoniae* exhibited ERV MIC value at a level of ≤2 mg/L (326/393) [14]. However, a study in 2023 reported a downward susceptibility rate of ERV in *K. pneumoniae* (53.1%) [34], along with the present study, indicating a potential emergence of ongoing resistance against ERV among *K. pneumoniae* in China. In addition, our study confirmed that mutations in efflux pump regulator genes (*ramR*, *oqxR*, and *acrR*) would contribute to not only ERV but also other commonly used antimicrobials resistance in *K. pneumoniae* through upregulating efflux pumps. To the best of our knowledge, this is the first study to testify to the function of several novel point mutations in *ramR* and prove their dual abilities in elevating drug resistance and enhancing host immune activation in *K. pneumoniae* simultaneously.

The role of the RND‐type efflux pump AcrAB‐TolC and quinolone/olaquindox efflux pump OqxAB played in the resistance mechanism of *K. pneumoniae* against tigecycline has been studied comprehensively [16, 18, 35, 36, 37, 38]. However, the key mutation points leading to it in *K. pneumoniae* are still not quite clear. Similar to tigecycline, the overexpression of AcrAB‐TolC/OqxAB will result in ERV resistance in *K. pneumoniae* [14, 29]. Exposure to EPI PAβN significantly reversed resistance against ERV in 97.6% of strains in our study, and even ERV‐susceptible strains can be affected by PAβN. This indicated that the influence of AcrAB‐TolC/OqxAB widely existed in *K. pneumoniae*. In addition, although strains in our study had not been exposed to ERV before, the resistance rate was considerably high (25%). We hypothesized that this may be due to the cross‐resistance between ERV and other antimicrobials which are also transported by these efflux pumps. This situation was reported both in tigecycline‐ and ERV resistance in *K. pneumoniae* [14, 16]. The findings in the present study may draw attention to rational antimicrobial use.

In this study, we found that AcrAB‐TolC played a more crucial role in ERV resistance compared to OqxAB. Some ERV‐susceptible strains showed significant expression of *ramA/oqxB/acrB*, suggesting other unknown downregulation pathways may be involved. Point mutations *ramR* I141T upregulated *ramA*, *acrB*, and *oqxB*, but ERV MIC values remained below the resistance criterion. The reason is that the *ramR* I141T mutation may not significantly impact RamR's function or the DNA‐binding affinity required to suppress *ramA* expression (proved by EMSA in this study). While the *ramR* L58P and F165L substitutions alter the secondary structure and disrupt interactions within the dimer interface (detailed RamR structural analysis in Supporting Information Text). Further investigation is needed to explore these mechanisms.

The key finding of our study is the confirmation of the functional significance of *ramR* L58P and F165L mutations in modulating host‐bacteria interactions and elevating resistance against ERV and other antimicrobials in *K. pneumoniae*. Prior research has highlighted the role of RamA in lipid A biosynthesis activation, leading to reduced microbial clearance and increased systemic dissemination of *K. pneumoniae* [28], and the AcrAB contributes to the survival of *Escherichia coli* in the macrophage environment [23]. Similarly, studies in *Enterobacter cloacae* demonstrated lower bacterial loads in mice spleens infected with *acrA* knockout strains [39]. In contrast to previous studies involving whole gene alterations, our study unveils two novel point mutations, *ramR* L58P and F165L, exhibiting a dual function in both ERV resistance and pathogenesis in *K. pneumoniae*. We observed that *ramR* L58P and F165L mutants induce an enhanced immune response, thereby reducing the anti‐killing ability of the pathogen in organs and blood. Our findings suggest that the functional loss of RamR, resulting from missense mutations in *ramR*, leads to increased *ramA*/*acrB* expressions, influencing *K. pneumoniae* lipid A composition and ultimately affecting its pathogenicity.

Lipid A, a critical component of gram‐negative bacteria virulence factors, is primarily recognized by TLR4/MD2 [40]. Altering the structure of lipid A modifies the host‐bacteria interaction and consequently impacts the pathogenesis of *K. pneumoniae* [32, 41, 42, 43]. Previous studies confirmed *lpxL_2*'s role in introducing a secondary myristate (C14) at the 2ʹ position of the lipid A structure in *B. pertussis* [44] and *K. pneumoniae* [45]. Notably, *lpxL* and *lpxM* are not essential in lipid A biosynthesis, but they exert a profound effect on the lipid A structural alteration and fitness of the bacteria [30]. In the present study, *ramR* L58P and F165L mutants showed overexpressed *lpxL*, *lpxL_2*, and *lpxM* and an elevated proportion of hydroxy lipid A. Further studies are required to validate the role of highly expressed genes involved in lipid A modification in *K. pneumoniae* in this work.

The relationship between lipid A and virulence has been studied in different bacteria and results are somehow contradicted. A previous study found that the deletion of *lpxL_2* increases the human phagocyte‐mediated killing of *K. pneumoniae* and the inflammation upregulation of inflammatory responses in macrophages upon infection [45]. In *Salmonella* and *Bordetella bronchiseptica*, a reduced potency to activate inflammatory reactions was detected in strains that inactivated some lipid A modification genes [44, 46]. An attenuated macrophage adhesion and up‐take were observed in RamA‐increased *K. pneumoniae* and it is linked to lipid A modification [28]. Usually, the number of lipid A acyl chains directly correlates with the ability to induce cytokine production, whereas the hexa‐acylated forms are usually the most immunostimulating ones [47]. Our findings challenge the conventional belief that lipid A modifications lead to reduced recognition by TLR4, thereby limiting inflammation and allowing pathogens to establish chronic infections in vivo. Our evaluation of cytokine levels secreted by macrophages and human blood supports the notion that *ramA*‐mediated lipid A modification can induce high levels of inflammation. Additionally, the acute reduction in organ bacterial burdens in mice infected with *ramR* L58P and F165L mutants further supports the occurrence of severe inflammation shortly after injection, triggering the host's innate immune response to clear the bacteria.

Macrophages are the primary effector cells of the host's innate immune system, responsible for phagocytosing bacteria, releasing cytokines and chemokines, and presenting antigens to trigger adaptive immune responses. Hypervirulent *K. pneumoniae* (hvKP) can be resistant to phagocytosis and intracellular killing by immune cells [48]. However, our study revealed that in addition to activating cytokine release in macrophages, lipid A structural modifications reduced bacterial survival within these cells. Consequently, *K. pneumoniae* with lipid A containing more hydroxylated fatty acid chains cannot disseminate throughout the host's body during macrophage migration and cannot persist in organs long‐term. Furthermore, due to the increased levels of stimulating cytokine production, bacteria with these mutations diminished the pathogenicity of hvKP which displayed weakened resistance against blood/serum bactericidal activity.

Besides antimicrobial agents, efflux pumps can also recognize antimicrobial peptides, dyes, and biocides, which helps bacteria become more adaptive in different environments [49, 50]. Several studies also showed that efflux pumps AcrAB can influence the virulence of gram‐negative bacteria. Our work supported this finding and further elucidated that changed *ramA* expression can modify lipid A structure and alter host‐bacteria interaction [23, 27]. Our study also showed that the biofilm formation ability was highly increased in *ramR* L58P and F165L mutants. This result indicates that genes associated with biofilm formation could also mediated by the *ramR*‐*ramA* system in *K. pneumoniae*. Our research has some limitations. While our study primarily focuses on key point mutations in *ramR*, which significantly impact *K. pneumoniae's* resistance to ERV, we acknowledge that a comprehensive omics analysis could provide a more extensive understanding of the regulatory networks and resistance mechanisms. Future studies incorporating transcriptomics, proteomics, or metabolomics would be valuable for uncovering additional factors and pathways through which *ramR* L58P and F165L activate the immune response, and further studies are needed to determine the primary immune cells affected in the bloodstream. Additionally, while *ramR* I141T did not exhibit lipid A structural changes, it showed increased virulence, and its underlying mechanism requires further investigation.

## 4 CONCLUSION

Our study reveals the profound significance of *ramR* mutations induced RamA‐mediated regulation in *K. pneumoniae*. We demonstrate that *ramR* plays a dual role in *K. pneumoniae*, protecting antimicrobial challenges and highly stimulating the host's innate immune response. Notably, our findings underscore the broader implications of *ramA* overexpression, as it leads to increased *acrAB* expression and lipid A alterations, culminating in decreased susceptibility to the newly invented last‐line drug ERV. Additionally, our study highlights the critical role of regulation proteins like RamR in regulating survival strategies of *K. pneumoniae* and potentially other *Enterobacteriaceae*, particularly in the context of microbial populations adapting to drug and host immune pressures. This raises concerns about selecting for *ramR* mutations not only in *K. pneumoniae* but also in other members of *Enterobacteriaceae*.

## 5 METHODS

### 5.1 Bacteria strains and growth conditions

The *K. pneumoniae* strains in this study were continuously collected from non‐duplicate clinical samples in five hospitals (Peking Union Medical College Hospital, Sir Run Run Shaw Hospital, Peking University First Hospital, Huashan Sub‐Hospital of Fudan University, The First Affiliate Hospital of Guangzhou Medical University) in China during 2016–2020 (Table S4), with each hospital 100 isolates. For the further investigation of the resistance mechanism, 119 strains were selected across a concentration gradient from 0.125 to 16 mg/L to encompass the MIC distribution profile for ERV (9 isolates for MIC = 0.125 mg/L, 18 for MIC = 0.25 mg/L, 8 for MIC = 0.5 mg/L, 32 for MIC = 1 mg/L, 24 for MIC = 2 mg/L, 12 for MIC = 4 mg/L, 15 for MIC = 8 mg/L, and 1 for MIC = 16 mg/L). All strains were sent to Peking Union Medical College Hospital for re‐identification by MALDI‐TOF MS (Autof MS1000, Autobio) by selecting a single colony from plated culture media and transferring it onto a MALDI target plate. Then overlie it with 1 μL matrix solution α‐cyano‐4‐hydroxycinnamic acid and allow it to air‐dry. Load the target plate into the MALDI‐TOF MS instrument, and use a laser power of 50%–70%, an ion source voltage of 20 kV, a mass range of 2000–20,000 Da, and 240–500 laser shots per spot to collect MS spectrums. Compare them against a microbial database. When identified as *K. pneumoniae*, isolates were cultured in China Blue Agar plates at 35°C.

### 5.2 Mice and ethics statement

Six‐week‐old female BALB/c mice were purchased from Beijing Vital River Laboratory Animal Technology Co., Ltd. Mice were raised in the Tsinghua University Animal Biosafety Level 2 (ABSL‐2) Laboratory for 1 week to adapt to the environment before being used as a model to analyze bacterial infection. The Institutional Animal Care and Use Committee of the Laboratory Animal Resources Center at Tsinghua University approved the animal experiments.

### 5.3 Macrophage cell line and culture conditions

The RAW264.7 mouse macrophage cell line was obtained from the Cell Resource Center at the Institute of Basic Medical Sciences (Beijing, China) and cultured in Dulbecco's modified Eagle's medium (DMEM) (Thermo Fisher Scientific) with 10% (v/v) fetal bovine serum (FBS) (Thermo Fisher Scientific) at 37°C with 5% CO_2_.

### 5.4 Determination of MIC

The ERV MIC was determined in Cationadjusted Mueller‐Hinton broth (CAMHB) following the Clinical and Laboratory Standards Institute (CLSI) guidelines with a susceptible breakpoint of ≤0.5 mg/L according to the FDA criteria. MICs of other antimicrobial agents (tetracycline, tigecycline, minocycline, colistin, cefoxitin, aztreonam, cefepime, ceftazidime, meropenem, ertapenem, ciprofloxacin, piperacillin/tazobactam, ceftriaxone, ceftolozane/tazobactam, imipenem, imipenem/relebactam, ceftazidime/avibactam, amikacin, and levofloxacin) were determined using the CLSI broth microdilution (BMD) method [51]. Clinical breakpoints from CLSI 2021 [51] were used for susceptibility interpretations. Quality control strains included *Escherichia coli* ATCC 25922, *Pseudomonas aeruginosa* ATCC 27853, and *Staphylococcus aureus* ATCC 29213.

### 5.5 PCR and gene sequencing

To examine the efflux pumps related genes and their mutations, efflux pump regulator genes *ramR*, *acrR*, *oqxR* and tetracycline‐resistant gene *tet(A)*, *tet(M)*, *tetL*, *tetX*, and ribosomal protein S10 gene *rpsJ* were amplified by PCR using specific primers listed in Table S5 and subsequently sequenced using Sanger sequencing. CLC sequence viewer 8.0 was applied to analyze the sequencing results. *K. pneumoniae* ATCC 13883 was used as a reference strain to define whether there was any mutation in tested strains.

### 5.6 Efflux pump inhibition test

To identify the role of the efflux pump in ERV resistance, the activity of efflux pumps was evaluated by EPIs phenyl‐arginine‐β‐naphthylamide (PAβN, Sigma). The ERV MIC was detected by BMD method with or without EPIs (50 µM), and each experiment was repeated 3 times. A fourfold change or more decrease in MIC values with the presence of EPIs was defined as a significant inhibition effect of EPIs [52].

### 5.7 RNA extraction and gene expression test

To evaluate whether efflux pump genes were overexpressed under regulator gene mutation, we detected transcription levels of efflux pump genes (*oqxB, acrB*), transcriptional regulator gene (*ramA*), and lipid A biosynthetic pathway gene (*lpxX*) using RT‐PCR. Total RNA was extracted from logarithmic growth phase bacteria with RNA pure Bacteria Kit (DNase I, CoWin Biosciences), and complementary DNA was synthesized using FastKing RT Kit (With gDNase, TIAGEN) from 1 µg of total RNA. qRT‐PCR was performed using TB Green Premix DimerEraser^TM^ (TaKaRa) in LightCycler 480Ⅱ (Roche) with primers from Table S5. Gene expression levels were normalized to *K. pneumoniae* housekeeping gene *rpoB*. The 2^−ΔΔCt^ method was used to compare target gene expression with that of *K. pneumoniae* ATCC 13883.

### 5.8 Construction of *ramR* point mutated strains

To conduct the functional validation of target gene mutations, we introduced point mutations (*ramR* L58P, I141T, and F165L) into *K. pneumoniae* AZJ065 strain (a CR‐hvKP with an ERV MIC of 0.0625 mg/L) using CRISPR‐Cas9 system with pCasKP‐apr and pSGKP‐spe plasmids. A two‐step process using a linear donor sequence and the spacer‐introduced pSGKP‐spe plasmid. Successful point mutations were confirmed by PCR and sequencing after curing the pCasKP and pSGKP plasmids. MICs and efflux pump activity of ERV were evaluated in the resulting colonies. Methods for competent cell preparation, electroporation, and spacer cloning followed Wang et al. [53]. Primers used in this study are listed in Table S5. The detailed process and whole genome sequencing result of three mutants and the WT were shown in Supplementary Text.

### 5.9 Protein expression and purification

The ORF of RamR was constructed on the pET15a vector (the same approach was taken for the RamR mutants). All constructs were verified by DNA sequencing (Beijing Ruibio BioTech Co., Ltd). The recombinant plasmid was used to transform *E. coli* BL21 (DE3) (TransGen Biotech). The cells were grown in LB medium, supplemented with 100 µg/mL ampicillin, at 37°C, 220 rpm. The culture was incubated at 37°C, 220 rpm until its OD_600_ had reached 0.6. The expression of N‐terminally hexahistidine tagged RamR was induced by 200 µM of isopropyl 1‐thio‐b‐d‐galactopyranoside (IPTG) and the culture was further incubated at 18°C, 220 rpm for an additional 16 h. After harvesting cells by centrifugation (5000 rpm, 15 min), the cells were resuspended in lysis buffer containing 50 mM Tris‐HCl pH 7.5 and 250 mM NaCl. The suspension was supplemented with 0.1 mM phenylmethylsulfonyl fluoride, 0.05 mg/mL lysozyme, 1 µg/mL DNaseI, and 10 mM MgCl_2_, followed by cell disruption using a high‐pressure homogenizer. Insoluble matter was removed by centrifugation at 4°C, 13,000 rpm for 50 min. The supernatant fraction was incubated with Ni‐NTA agarose (QIAGEN) that had been pre‐equilibrated with lysis buffer. The column was washed with 50 mM of Tris‐HCl pH 7.5, 500 mM of NaCl, and the RamR‐containing fraction was eluted in 50 mM of Tris‐HCl pH 7.5, 250 mM of imidazole pH 8.0. The protein was further purified by ion exchange chromatography and size exclusion chromatography. RamR and its mutant proteins were finally stored in phosphate‐buffered saline (PBS) buffer at −80°C.

### 5.10 EMSA assay

The DNA probe used for EMSA, which contained the RamR–ramA intergenic region was purchased from Beijing Ruibio BioTech Co., Ltd. A total volume of 40 µL EMSA reaction mixture contained 200 nM dsDNA (pre‐annealing) and 2 µM purified RamR (or its mutants) in the binding buffer (0.5 × TBE buffer). After incubating 15 min at room temperature, the samples were loaded onto 5% Non‐denaturing Pre‐cast Gel (Shanghai Wansheng Haotian Biotechnology Co., Ltd.) and electrophoresed 120 V 40 min in 0.5 × TBE buffer and finally stained with NA‐Green (a fluorescent DNA dye, Beyotime Co., Ltd.). The bands were visualized with Typhoon NIR (Cytiva).

### 5.11 Biofilm detection

To evaluate the biofilm [54] formation ability, mutants, WT, *K. pneumoniae* ATCC 13883, and *K. pneumoniae* ATCC 700603 were cultured overnight on Blood Agar plates. Samples were then seeded at 5 × 10^7^ CFU/mL with eight repeats in 96‐well plates and incubated at 37°C for 48 h. Biofilms were stained with 1% crystal violet for 15 min, washed with deionized water, and then measured at 590 nm after resuspension in 96% ethanol.

### 5.12 Lipid A extraction and analysis

To examine the impact of *ramR* mutations on the lipid A moiety, the lipid A extraction [55] was displayed as before. Bacteria were cultured on Blood Agar plates, and one colony was selected for overnight culture in 5 mL LB broth. Cells were suspended in 1 mL LB broth after centrifugation at 5000 rpm for 12 min. Samples were heated at 80°C for 1 h, collected by centrifugation at 1000 rpm for 3 min, and washed three times with double‐distilled water. Cells were then resuspended in 200 μL double‐distilled water, treated with 200 μL 2% acetic acid, and incubated at 100°C for 30 min. After three more washes with double‐distilled water, cells were resuspended in 100 μL double‐distilled water. The bacterial solution (0.4 μL) was mixed with Super‐2,5‐dihydroxybenzoic acid substrate (0.8 μL) on the sample target and dried. Analysis was performed using a 4800 Proteomics Analyzer mass spectrometer at 20 kV in negative ion mode with a 20 ns extraction delay time. Data was analyzed with Data Explorer software (Version 4.9).

### 5.13 Growth curve assays

To determine whether lipid A species abundance changes will alter *K pneumoniae* growth rate, WT strain AZJ065, and three mutants were cultured overnight in LB broth. Samples were then seeded at 10^6^ CFU/mL in quadruplicate in 96‐well plates and incubated at 37°C in a microplate reader (BioTek EPOCH2, Agilent Technologies Inc.). Absorbance (Optical Density at 600 nm, OD_600_) values were recorded every 30 min for 24 h. Growth rate (μ) was calculated from the exponential segment of the growth curve using ln 2 g^−1^, where g is the time for exponential growth to double in size [56] and was calculated using R 4.2.1.

### 5.14 Mouse infection model

To evaluate the pathogenicity of mutants, bacteria cultured in LB broth to exponential phase were stored at −80°C. Before the experiment, bacteria were collected by centrifugation at 6000 rpm for 6 min, washed once with PBS, and resuspended in the same buffer. For survival rate determination, mice were intraperitoneally injected with three mutants and WT at 5 × 10^7^ CFU/mL (100 μL per mouse, five mice per group). Bacteria clearance assays were conducted by intravenous injection at 1 × 10^7^ CFU/mL (100 μL per mouse, six mice per group) at multiple time points (0.5, 1, 1.5, 2, 3, and 6 h). Blood samples were collected for bacteria burden calculation. Organ bacteria burden was determined by sacrificing mice at 6, 12, 24, and 48 h post‐infection (five mice per group, i.p.). Spleen, liver, and lung samples were harvested, homogenized in PBS, and serially diluted for bacteria burden calculation.

### 5.15 *ramR* mutations contribution to ERV resistance

To confirm the *ramR* mutations’ contribution to ERV in vitro, mice were intramuscularly injected with 1 × 10^7^ CFU/mL bacteria (100 μL per mouse, three mice per group) into the posterior thigh muscle. A control group received 100 μL PBS injection. ERV was administered intravenously at a loading dose of 10 mg/kg, 2 h after infection, and every 12 h thereafter. Mice were euthanized after 72 h. Thighs were individually homogenized in 1.2 mL PBS, and CFU determination was performed by plating serial dilutions of the homogenate onto LB agar.

### 5.16 Anti‐whole blood/serum killing assay and cytokines analysis

To evaluate the innate immune level induced by mutants, bacteria were cultured in 5 mL LB medium at 37°C, 180 rpm, and diluted to exponential phase (OD_600_ = 0.3). After washing with PBS, 5 × 10^7^ CFU/mL bacterial suspensions were prepared. Fresh human blood and serum (used within 30 min) were mixed with 5 × 10^5^ CFU bacteria and incubated at 37°C, 180 rpm. Viable counts were obtained after 0, 1.5, and 3 h incubation. Experiments used blood from healthy donors, with each strain tested in triplicate. Cytokines (IL1β, IL2, IL4, IL5, IL6, IL8, IL10, IL12p70, IL17, TNF‐α, IFN‐α, and IFN‐γ) were detected using EasyMagPlex Human Cytokine 12 Plex Kit (Shenzhen Wellgrow Technology Inc.) in the department of clinical laboratory at Peking Union Medical College Hospital.

### 5.17 Macrophage phagocytosis assay

To test the macrophage activation capacity of *ramR* mutants, RAW264.7 cells (5 × 10^5^ cells/well) were cultured in 12‐well plates with DMEM (10% FBS) for 16 h. Fresh DMEM with bacteria (2.5 × 10^7^ CFU/mL, multiplicity of infection (MOI) = 20) was added for 2 h. After washing with PBS, DMEM (containing 1 mg/mL apramycin) was added for 2 h to kill extracellular bacteria. Intracellular bacteria were quantified by plating lysates onto LB agar plates. Survival ability inside macrophages (MOI = 100) was assessed by incubating cells further in DMEM (10% FBS) and quantifying intracellular bacteria at different time points. Cytokine levels (IL1β, IL6, and TNF‐α) were examined in supernatants using the Mouse IL1β/IL6/TNF‐α ELISA Kit (MULTI SCIENCES).

### 5.18 Statistical analysis

SPSS 26.0 (IBM) performed all statistical analyses. Kruskal–Wallis H analysis was used for non‐normally distributed data. Linear Regression analyzed the relation between mutations in *ramR, acrR, oqxR*, and ERV MIC in *K. pneumoniae*. GraphPad Prism 8, with Log‐rank test (Mantel‐Cox), analyzed the survival curve in the mouse infection model. Data following a normal distribution were analyzed using *t*‐test or analysis of variance, and *p* values of <0.05 were used to denote statistical significance.

## AUTHOR CONTRIBUTIONS

**Qiwen Yang**: conceived and designed the study. **Wei Yu, Xiaobing Chu, Peiyao Jia, Shengjie Li, Xinmiao Jia, Ying Zhu, and Xiaoyu Liu**: performed the experiments and analyzed the data. **Wei Yu and Peiyao Jia**: prepared the manuscript. **Yingchun Xu and Qiwen Yang**: revised the manuscript. All authors have read the final manuscript and approved it for publication.

## CONFLICT OF INTEREST STATEMENT

The authors declare no conflicts of interest.

## ETHICS STATEMENT

The study protocol was reviewed by the Human Research Ethics Committee of the Institutional Review Board (IRB) of the Peking Union Medical College Hospital (ethics approval number S‐K 1253). This project did not affect the routine diagnosis and treatment of patients; after consultation with the IRB, formal ethical approval was reviewed and waived, and written patient consent was not required. The ethics application (No. 23‐ZJR‐1) was approved by the Research Ethics Committee of the University of Tsinghua.

## Supporting information


**Figure S1:** Efflux pump inhabitation test panels.
**Figure S2:** Fitness cost of *K. pneumoniae* harboring target mutations naturally.
**Figure S3:** Structure of RamR dimer.

**Table S1:** Eravacycline MIC with or without PAβN, gene mutations of *ramR*, *oqxR*, and *acrR*, and gene expression level of *ramA*, *oqxB*, and *acrB* of 119 isolates.
**Table S2:** Gene mutation types in 119 *K. pneumoniae* clinical isolates.
**Table S3:** Linear Regression of gene factors related to MIC.
**Table S4:** Information of 500 *Klebsiella pneumoniae* isolated from 5 hospitals.
**Table S5:** Primers used in this study.

## Data Availability

The data that supports the findings of this study are available in the supplementary material of this article All data generated or analyzed during this study are included in this published article and its supplementary information files. The information on *Klebsiella pneumoniae* AZJ065 can be obtained from accession number: CP099519. The data and scripts used are saved in GitHub: https://github.com/supertermial/Dual-role-of-ramR-mutation_iMeta. Supplementary materials (methods, results, figures, tables, graphical abstract, slides, videos, Chinese translated version, and update materials) may be found in the online DOI or iMeta Science http://www.imeta.science/imetaomics/.
