# Supplementary material for: Dual role of ramR mutation in enhancing immune activation and elevating eravacycline resistance in Klebsiella pneumoniae
Source: IMetaOmics. 2024 Nov 9;1(2):e39. doi: 10.1002/imo2.39 (PMC12806483; doi:10.1002/imo2.39)
Supplement: Supplementary file 1 — Figure S1: Efflux pump inhabitation test panels. Figure S2: Fitness cost of K. pneumoniae harboring target mutations naturally. Figure S3: Structure of RamR dimer. [file IMO2-1-e39-s001.docx]

**Supplementary information to**

**Dual role of *ramR* mutation in enhancing immune activation and elevating eravacycline resistance in *Klebsiella pneumoniae***

**Running title:** *ramR* mutation enhancing immune activation and eravacycline resistance

Wei Yu^1,2#^, Peiyao Jia^1#^, Xiaobing Chu^1,2#^, Shengjie Li^1,3#^, Xinmiao Jia^1,3^, Ying Zhu^1,2^, Xiaoyu Liu^1^, Yingchun Xu^1^, Qiwen Yang^*1,4^

^1^Department of Clinical Laboratory, State Key Laboratory of Complex Severe and Rare Diseases, Peking Union Medical College Hospital, Chinese Academy of Medical Sciences and Peking Union Medical College, Beijing 100730, China

^2^Graduate School, Peking Union Medical College, Chinese Academy of Medical Sciences, Beijing 100730, China

^3^Medical Research Center, State Key Laboratory of Complex Severe and Rare Diseases, Peking Union Medical College Hospital, Chinese Academy of Medical Sciences and Peking Union Medical College, Beijing 100730, China

^4^Key Laboratory of Pathogen Infection Prevention and Control (Peking Union Medical College), Ministry of Education, Beijing 100730, China

^#^These authors contribute equally: Wei Yu, Peiyao Jia, Xiaobing Chu, Shengjie Li

^*^Correspondence: yangqiwen81@vip.163.com (Qiwen Yang)

**Supplementary methods**

**Construction of *ramR* and *acrR* point mutated strains**

The point mutation was constructed in *Klebsiella pneumoniae* AZJ065 strain (the MIC of eravacycline was 0.0625 mg/L and harbored wide-type AcrAB-TolC /OqxAB efflux pump genes) using CRISPR-Cas9-mediated genome-editing method containing pCasKP-apr and pSGKP-spe plasmid. A two-step genome editing procedure was applied to construct mutations of *ramR* L58P, I141T, F165L, and *acrR* M1I. Firstly, ~500 bp sequences were deleted up and downward the point mutation by co-transforming a linear donor sequence and the spacer-introduced pSGKP-spe plasmid, individually, into the L-arabinose-induced pCasKP-apr-harboring cells to partially delete the target sequence. After gene partial deletion, the pSGKp plasmid was cured by culturing in the LB agar plate containing 50 mg/L apramycin and 5% sucrose at 30 °C. Secondly, the sequence containing point mutation was complemented into the cells using pSGKP which spacer was located in the interfaces for upstream and downstream sequences of deleted sequences in the first step. The MICs and efflux pump activity of eravacycline were evaluated in the colony in which successful point mutation was confirmed by both PCR and sequencing after pCasKP and pSGKP plasmid curing. Preparation of competent cells, electroporation, and Spacer cloning into pSGKP plasmid was performed as Wang Y et. described^1^. The primers used in this study are listed in Table S1.

**Supplementary results**

**Whole-genome sequencing of 4 mutants and the WT**

We performed whole-genome sequencing on four mutant strains and one wild-type strain, and after comparing the sequences, we found that aside from point mutations in the target gene, there were no synonymous mutations in the genomes of the four mutant strains. Due to the quality of sequence assembly, we are unable to present the gene comparison results in a circular diagram. The genomic data of the five strains will be uploaded as an attachment.

***K. pneumoniae* survived under 50 μM PAβN**

In the evaluation of efflux pump inhibition, a control sample containing only Luria-Bertani (LB) broth and 50 μM Phenylalanine-arginine β-naphthylamide (PAβN) was prepared (Figure S1). Of the 119 strains tested, five (E02-348, E04-112, E04-115, E04-196, E05-197) exhibited no growth in the presence of PAβN and were thus omitted from subsequent analyses investigating the correlation between genetic mutations and eravacycline resistance. Additionally, strains that naturally harbored target mutations were assessed to determine the fitness cost associated with PAβN exposure. Statistical analysis revealed no significant difference in growth except E05-141 (*ramR* I141T K194*) and E04-191 (*ramR* F165L). Although the two strains exhibited a reduced growth rate in the presence of PAβN, they ultimately reached a comparable final biomass as those cultivated in LB broth (Figure S2 A-H). Furthermore, engineered mutants derived from the ZJ65 strain demonstrated viability upon the addition of PAβN (Figure S2I).

**Structural modification caused by *ramR* mutations**

The alpha-8 and alpha-9 helix account for the majority of the buried surface area between the dimer. Amino acid F165 is located on alpha-8 and points towards another monomer of the dimer, which may mediate hydrophobic interactions (Figure S3). The amino acid substitution from F to L has a significant difference in the side chain size. This change may lead to the expansion of the relative distance of the DNA-binding motif, a similar manner that is induced by the liganded RamR. This DNA-binding motif movement may contribute to the reduction in RamR DNA-binding affinity required to depress ramA expression in response to multidrugs. The side chain of I141 points to the outer space of the dimer, the point mutant in this site may have a minor influence on the RamR function.

The introduction of a proline residue into an alpha helix is likely to disturb the secondary structure and create a distinctive "kink" because of its rigidity and constrained phi angle. Amino acid residues L58 locate in the N-terminal of the alpha-4 helix, which is adjacent to the helix-turn-helix DNA binding motif. Thus the L58P amino acid substitution could potentially impact the DNA-binding affinity of RamR, resulting in increased expression levels of *ramA* and *acrAB*.

**Reference**

1. Wang,Yu, Shanshan Wang, Weizhong Chen, Liqiang Song, Yifei Zhang, Zhen Shen, Fangyou Yu, Min Li, Quanjiang Ji. 2018. “CRISPR-Cas9 and CRISPR-Assisted Cytidine Deaminase Enable Precise and Efficient Genome Editing in *Klebsiella pneumoniae*.” *Applied and Environmental Microbiology* 84: e01834-18. https://doi.org/10.1128/AEM.01834-18

**Supplementary figures**

**Figure S1 Efflux pump inhabitation test panels. The last columns are control samples containing only LB broth and 50 μM PAβN. The presence of turbidity in the liquid or bacterial colonies indicates that the strain can grow.**

**Figure S2 Fitness cost of *K. pneumoniae* harboring target mutations naturally.** (A-E) Fitness cost of strains harboring *ramR* I141T. (F) Fitness cost of strain harboring *ramR* L58P I141T. (G) Fitness cost of strain harboring *ramR* F165L. (H) Fitness cost of strain harboring *acrR* M1I. (I) Efflux pump inhabitation test panel of 4 engineered mutants. The last columns are control samples containing only LB broth and 50 μM PAβN.

**Figure S3 Structure of RamR dimer.**
